# Trends in exposures to physically demanding working conditions in France in 2003, 2010 and 2017

**DOI:** 10.1093/eurpub/ckab195

**Published:** 2021-11-11

**Authors:** Nathalie Havet, Alexis Penot

**Affiliations:** Laboratoire SAF, Université Claude Bernard Lyon 1, Lyon, France

## Abstract

**Background:**

To explore trends in social and occupational inequalities in terms of exposures to physically demanding working conditions for French employees.

**Methods:**

Our study assessed data from the French national cross-sectional survey of occupational hazards (SUMER) that was conducted in 2003, 2010 and 2017. Trends in the prevalence of several types of physically demanding working conditions (lifting of heavy loads, awkward postures, vibrations, harmful noise, extreme temperatures, and carcinogenic, mutagenic and reprotoxic agents) were explored. Temporal changes in associations of individual and job characteristics with these factors of hardship at work were examined using multilevel logistic regressions.

**Results:**

We estimated that 53.5% of French workers from all industries in the private sector and in public hospitals were exposed to at least one of the adverse physical working conditions considered in 2017. While the prevalence of exposure to severe physical constraints increased between 2003 and 2017 (+4.2 pp), the exposure associated with a hazardous physical environment decreased sharply (−6.3 pp). These observed trends did not occur similarly for all workers. Several inequalities in exposure increased over the period, particularly to the detriment of blue-collar workers. The situation of shift workers deteriorated in terms of the exposure to vibrations and awkward postures.

**Conclusion:**

Our study indicates that more stringent interventions are needed to reduce the prevalence of pronounced physical constraints that contribute to MSDs. Future prevention strategies, in addition to seeking to achieve a general reduction in exposure to all physically demanding working conditions, should aim to reduce disparities that adversely affect vulnerable populations.

## Introduction

In recent decades, public authorities have become increasingly aware of the importance of reducing occupational hazards, and this has led to the implementation of general public health policies in Europe and multiple EU directives for occupational hazard prevention (https://osha.europa.eu/en/safety-and-health-legislation/european-directives), such as the REACH regulations on harmful chemical exposures. France has of course followed this trend in its national policy. For example, the objective of the French *Law on public health policy* (2004) was to improve not only the prevention of occupational diseases but also their recognition and treatment. Thus, the *Occupational Health Plan (PST1) 2005-2009* reformed the national occupational risk prevention system by the introduction of more stringent means of intervention and control of the labor administration and inspection. The subsequent *Occupational Health Plan 2010-2014* had the general objective of strengthening the prevention of occupational accidents and diseases, while also reducing exposure to these risks. To do this, one of the main stated goals was the development of actions to prevent occupational risks, in particular, to limit chemical risks and musculoskeletal disorders (MSDs). At the same time, the focus of reducing exposure to carcinogenic, mutagenic and reprotoxic (CMR) substances in the workplace was included in the *National Health-Environment Plan 2009-2013.* Finally, the *French Cancer Plan 2009-2013* in fact specifically stated the objective of reducing the number of employees exposed to CMR products by 100 000 in the 2009–12 timeframe.

In light of these regulatory measures, we wished to analyze changes in exposures to physically demanding working conditions (lifting of heavy loads, awkward body postures, vibrations, harmful noise, extreme temperatures and CMR agents) between 2003, 2010 and 2017, as well as to probe for inequalities in terms of exposures to these conditions. We wished to determine whether there was a discernible decrease in this period of time in the exposures to these occupational risks. Of particular interest, if such a decrease could be confirmed, was the question whether this occurred unequally and hence to the detriment of some employees for whom the exposure could in fact have increased.

## Methods

### Study population

The SUMER survey is a national cross-sectional survey that is conducted periodically by the French Ministry of Labor and the Directorate for Research, Studies, and Statistics (DARES) to assess occupational risks among a representative sample of the French employee population. The survey is based on a two-level sampling involving voluntary occupational physicians (1792 in 2003, 2400 in 2010 and 1243 in 2017) who randomly selected employees over a 3-month period (56 245 in 2003, 53 940 in 2010 and 33 600 in 2017) among those who had undergone their mandatory periodical medical check-ups. Each physician (interviewer) was asked to undertake between 20 and 30 interviews and to complete a standardized questionnaire after obtaining the employee's consent. The response rates were approximately 80% for the three surveys. The physicians reported whether or not each employee was exposed to a specific list of various chemicals, biological agents and physical constraints over a period of 1 week, and for each physical constraint identified, they were asked to report the duration of exposure using a categorical variable: <2, 2–10, 10–20 or >20 h in a single workweek. They relied on statements provided by the employees and their knowledge of the field and the work processes that are specific to the company or the job. If in doubt, the physician could perform a more in-depth workplace assessment.

Although the 2003 SUMER survey was repeated in 2010 and in 2017 using the same methodology, two elements had to be taken into account to allow comparability over time. First, the target population covered by the SUMER survey was extended over the years. Thus, we restricted our analysis to the common scope of the two surveys, i.e. workers from all industries in the private sector and in public hospitals in metropolitan France (representing 80% of all French employees). Second, the list of the chemical agents was extended between 2003, 2010 and 2017 based on policy advancements and substitutions undertaken by companies, as well as a result of increased knowledge regarding toxicities.^1^ Nevertheless, for the CMR agents, the difference between the three surveys is limited to the family of glycol ethers (not assessed in 2003) and the carcinogenic pesticides (not assessed in 2003 or in 2010). Overall, we restricted our temporal analysis of the exposures to chemicals to the 27 CMR agents identified as being common to the three surveys.

Post-data collection adjustment accounted for the characteristics of occupational physicians, the periodicity of medical visits and the characteristics of non-respondents. The data were further weighted by industry sector on gender, age, nationality, working time, occupation, company size and economic activity to ensure the representativeness of the survey samples and exposure prevalences of the target populations in 2003, 2010 and 2017.

### The physically demanding working conditions considered

Using the SUMER survey, exposures to the following physical working conditions were analysed: (i) *lifting, holding* *and carrying of heavy loads*; (ii) *awkward body postures* (holding one’s arms above shoulder level, kneeling, and/or crouched position, neck constraints, etc.); (iii) *arm/hand vibrations* caused by machines (grinders, chainsaws, jackhammers, etc.); (iv) *harmful noise* (noise > 85 dB, impulse noise); (v*) extreme temperatures* (<15°C or >24°C imposed by the production process); and (vi) *exposure to CMR agents* (27 agents classified as being carcinogenic or probably carcinogenic or mutagenic in humans by the IARC or classified as known, presumed, or suspected to have CMR potential in humans according to the European Union regulations^2^^,^^3^).

The risk that exposure of a worker to a physical constraint will eventually become manifest as an adverse effect on their health depends, among other factors, on the duration of the exposure: the longer the exposure, the greater the risk. However, it is impossible to determine a threshold for the duration of exposure below which the health risk would be negligible. Here, we considered thresholds—definable with the SUMER survey—above which physical working conditions could be classified as demanding due to their arduousness and significantly increased pathogenicity when these thresholds are exceeded. The chosen thresholds, which were similar to those in previous studies,^4–7^ were not particularly restrictive, yet nonetheless relevant in terms of prevention. They were 10 h/week for the lifting of heavy loads, extreme temperatures, noise >85 dB and 2 h/week for awkward postures, vibrations and impulse noise. For CMR agents, all exposures were considered irrespective of their duration.

### Statistical analysis

Descriptive statistics were used to examine trends in the prevalence of the various physically demanding working conditions. Associations of individual, job, and company characteristics with each exposure prevalence were studied using logistic regressions with random effects^2^^,^^8^^,^^9^ on the pooled sample created with the three SUMER editions. The economic activity of the company was modeled with a random intercept to account for heterogeneity in the exposure probability at a sufficiently disaggregated level. The covariates included were three variables describing employee characteristics (age, gender and seniority), five variables related to job characteristics (employment contract, work hours, work schedules, occupation and the main occupational duties) and two company characteristics (company size and geographical location). Two dichotomous variables identifying the edition of the survey and interaction terms between the explanatory variables and these dichotomous variables were introduced in the models to determine whether the determinants of the dependent variables changed over the period (2003, 2010 and 2017). In such specifications, the coefficients associated with the explanatory variables capture the effects of the reference year (2003)—they are called *main effects—*and the interaction terms capture the temporal changes from that reference year. More specifically, if for a given explanatory variable, the associated interaction term for 2010 (or 2017) was not statistically significant, it means that the effect of this variable on the dependent variable remained constant between 2003 and 2010 (or 2017). All of the analyses were performed using STATA V.16.0 software (StataCorp., College Station, TX).

## Results

### An increase in exposures to some physically demanding working conditions 

All up, we estimated that 53.4% of workers from all industries in the private sector and in public hospitals, corresponding to 9.8 million French employees in 2017, were exposed to at least one of the physical working conditions identified in the SUMER survey. This proportion decreased by three points between 2010 and 2017, returning to the level in 2003 (table 1). However, not all of the occupational risk exposures followed this general trend. While the prevalence of exposure to severe physical constraints increased from 44.6% to 48.8% (+4.2 points) between 2003 and 2017, exposure associated with a hazardous physical environment decreased sharply from 24.3% to 18% (−6.3 points) over the same period.

**Table 1:** Prevalence of exposure to physically demanding working conditions in France in 2003, 2010 and 2017

	SUMER (%)		
	2003	2010	2017
At least one of the physical working conditions	53.67	56.51	53.50
Severe physical constraints	44.61	49.42	48.80
Handling of heavy loads	13.24	11.30	8.51
Awkward body postures:	36.87	42.76	45.18
Holding one’s arms up	7.99	8.53	9.00
Kneeling	7.01	7.12	10.24
Neck constraints	19.68	25.66	20.83
Other postural constraints	16.13	15.88	27.56
Arm/hand vibrations	6.27	7.01	6.24
Hazardous physical environment	24.32	21.05	17.91
Harmful noise	12.70	12.29	9.52
Noise >85 dB	8.30	7.71	4.71
Impulse noise	7.41	7.56	6.80
Extreme temperatures	4.78	4.16	3.38
<15 °C	2.43	1.88	1.81
>24 °C	2.47	2.44	1.63
Exposure to CMR agents	14.15	10.89	9.78

The proportion of workers exposed to harmful noise, extreme temperatures and CMR agents declined continuously throughout the period. The prevalence of exposure to repeated handling of heavy loads (10 h/week or more) also declined sharply between 2003 and 2017 (from 13.2% to 8.5%). This decrease is mainly attributable to changes in the duration of the exposure. Indeed, the rate of exposure to carrying of heavy loads for any duration increased from 57% in 2003 to 64% in 2017. But among the exposed workers, the proportion with exposure durations of more than 10 h/week decreased from 31.6% to 25.8% over the same period.

The prevalence of exposure to awkward postures increased from 37% in 2003 to 45% in 2017, mainly due to increased exposures to kneeling, and squatting and twisting postures (other postural constraints), while the prevalence of exposure to vibrations remained stable.

### Changes in exposure inequalities

Multiple regressions (tables 2 and 3) show that the observed decreases in the prevalence of exposure to factors generating a hazardous physical environment and to the repeated handling of heavy loads did not occur to the same extent for all workers. Some workers experienced greater reductions. (They are identified by a significant OR and less than 1 for the 2017 interaction term.) Examples include workers with a specific status for exposure to harmful noise or extreme temperatures, employees working on Sundays for exposure to carrying of heavy loads or extreme temperatures, or employees in shift work or hired in medium-sized companies (50–499 employees) for exposure to CMR agents. In 2003, these groups of employees had higher probabilities of exposure to the factors considered, but the differences in exposure narrowed between 2003 and 2017. Moreover, employees in management and accounting duties were less likely to be exposed to harmful noise or the carrying of heavy loads in 2003, and this was even more so in 2017, as was also the case for administrative assistants, typists, and receptionists for the latter exposure.

**Table 2:** Results from multivariate random effect logit regressions on exposures associated with a hazardous physical environment (SUMER 2003, 2010 and 2017)

	Harmful noise			Extreme temperatures			CMR		
	Adjusted prevalence odds ratios^a^			Adjusted prevalence odds ratios^a^			Adjusted prevalence odds ratios^a^		
	(95% CI)			(95% CI)			(95% CI)		
	Main effect	Interc.	Interc.	Main effect	Interc.	Interc.	Main effect	Interc.	Interc.
		2010	2017		2010	2017		2010	2017
Men	2.35*** (2.12–2.59)	0.84** (0.72–0.97)	0.94 (0.77–1.14)	1.12 (0.99–1.27)	1.08 (0.89–1.30)	1.30*** (1.01–1.66)	1.76*** (1.61–1.92)	1.01 (0.88–1.16)	1.41*** (1.18–1.69)
Age	1.00** (0.99–1.00)	1.00 (0.99–1.00)	0.99 (0.99–1.00)	1.00 (0.99–1.00)	1.00 (0.99–1.00)	1.00 (0.99–1.01)	0.99*** (0.99–0.99)	0.99*** (0.99–1.00)	1.00 (1.00–1.01)
Job seniority (Ref: 10 years or more)									
<1 year	0.92 (0.79–1.07)	0.89 (0.72–1.11)	0.82 (0.60–1.12)	1.07 (0.87–1.32)	0.80 (0.59–1.09)	0.65 (0.42–1.02)	0.66*** (0.57–0.76)	1.04 (0.84–1.30)	1.14 (0.84–1.54)
1–3 years	0.96 (0.87–1.05)	1.02 (0.87–1.18)	0.99 (0.81–1.22)	0.93 (0.80–1.07)	1.02 (0.82–1.28)	1.01 (0.75–1.37)	0.85*** (0.77–0.92)	1.01 (0.88–1.17)	1.28** (1.05–1.55)
4–9 years	0.97 (0.89–1.05)	1.02 (0.90–1.15)	1.02 (0.88–1.20)	1.11 (0.98–1.25)	0.97 (0.80–1.16)	0.89 (0.70–1.12)	0.89*** (0.82–0.96)	1.00 (0.89–1.13)	1.23*** (1.06–1.42)
Full time	1.50*** (1.28–1.76)	1.04 (0.84–1.30)	1.39** (1.01–1.91)	1.46*** (1.22–1.76)	0.93 (0.72–1.22)	1.34 (0.90–1.98)	1.36*** (1.20–1.54)	0.91 (0.75–1.10)	0.86 (0.67–1.11)
Shift work	1.61*** (1.49–1.75)	0.99 (0.88–1.12)	1.07 (0.91–1.26)	1.34*** (1.20–1.51)	0.80** (0.67–0.95)	1.08 (0.86–1.37)	1.39*** (1.28–1.49)	0.88** (0.78–0.99)	0.85** (0.73–0.99)
Night work	1.28*** (1.18–1.39)	1.06 (0.93–1.20)	0.90 (0.76–1.05)	1.39*** (1.24–1.55)	0.95 (0.80–1.12)	0.93 (0.74–1.17)	1.13*** (1.05–1.22)	1.07 (0.95–1.21)	1.08 (0.93–1.26)
Sunday work	0.84*** (0.77–0.91)	1.00 (0.89–1.13)	1.24*** (1.06–1.45)	1.37*** (1.23–1.53)	1.02 (0.87–1.20)	0.47*** (0.38–0.59)	1.02 (0.95–1.10)	0.87** (0.78–0.98)	0.98 (0.85–1.14)
Employment contract (Ref: Permanent contract)									
Agency workers.fixed-term contract	1.24*** (1.09–1.42)	0.82** (0.69–0.98)	0.95 (0.73–1.23)	0.96 (0.80–1.15)	1.06 (0.82–1.37)	1.27 (0.88–1.81)	1.07 (0.95–1.22)	0.92 (0.76–1.10)	1.03 (0.80–1.33)
Workers with a specific status^b^	1.21 (0.93–1.57)	0.75 (0.56–1.00)	0.65** (0.45–0.92)	2.56*** (1.91–3.44)	0.26*** (0.18–0.38)	0.22*** (0.13–0.37)	0.79*** (0.67–0.94)	1.19 (0.96–1.47)	1.25 (0.96–1.64)
Occupational status (Ref: Technicians and associate professionals)									
Executives, managers	0.55*** (0.45–0.67)	0.96 (0.73–1.27)	1.00 (0.74–1.37)	0.30*** (0.21–0.44)	0.90 (0.54–1.51)	2.11*** (1.25–3.55)	0.48*** (0.41–0.55)	0.83 (0.66–1.04)	0.93 (0.72–1.20)
Clerks	0.71*** (0.55–0.91)	1.40 (0.99–1.98)	1.06 (0.62–1.81)	0.47*** (0.33–0.68)	0.98 (0.55–1.74)	0.94 (0.38–2.31)	0.46*** (0.37–0.57)	1.31 (0.94–1.82)	0.88 (0.51–1.52)
Service workers	0.81 (0.65–1.00)	1.11 (0.84–1.48)	1.10 (0.77–1.57)	0.85 (0.70–1.03)	1.29 (0.98–1.71)	1.11 (0.77–1.61)	0.65*** (0.56–0.75)	1.55*** (1.26–1.92)	1.59*** (1.22–2.08)
Skilled blue-collar workers	1.88*** (1.72–2.06)	1.15** (1.00–1.33)	1.20** (1.01–1.42)	1.66*** (1.43–1.93)	0.97 (0.78–1.22)	0.97 (0.73–1.28)	1.46*** (1.34–1.59)	1.28*** (1.12–1.46)	1.86*** (1.59–2.17)
Unskilled blue-collar workers	2.05*** (1.84–2.28)	1.18** (1.00–1.38)	1.02 (0.82–1.26)	2.05*** (1.73–2.43)	0.90 (0.70–1.15)	1.07 (0.78–1.48)	1.29*** (1.16–1.42)	1.31*** (1.12–1.53)	1.23 (0.99–1.53)
Main occupational duties (Ref: Production, Manufacturing and construction)									
Installation, repair, maintenance	0.98 (0.90–1.07)	0.96 (0.85–1.10)	1.18 (0.99–1.40)	0.48*** (0.41–0.57)	1.00 (0.78–1.27)	1.18 (0.85–1.62)	1.85*** (1.71–2.01)	1.02 (0.90–1.15)	0.99 (0.84–1.17)
Cleaning, childcare. home management	0.31*** (0.24–0.39)	0.99 (0.72–1.35)	1.79*** (1.20–2.68)	0.40*** (0.31–0.51)	0.99 (0.68–1.44)	0.96 (0.56–1.65)	0.40*** (0.33–0.50)	0.98 (0.72–1.34)	0.96 (0.62–1.48)
Handling, logistics, warehousing	0.22*** (0.20–0.25)	1.04 (0.87–1.24)	1.22 (0.95–1.57)	0.55*** (0.48–0.65)	1.09 (0.87–1.37)	1.23 (0.90–1.68)	0.32*** (0.29–0.36)	0.76*** (0.63–0.93)	0.96 (0.75–1.22)
Administrative assistant, typing, receptionist	0.15*** (0.11–0.21)	0.65 (0.39–1.07)	0.95 (0.47–1.93)	0.14*** (0.09–0.24)	1.05 (0.51–2.15)	1.04 (0.38–2.89)	0.19*** (0.14–0.25)	0.57** (0.35–0.95)	0.97 (0.50–1.89)
Management and accounting	0.12*** (0.10–0.16)	0.62** (0.38–1.00)	0.31** (0.11–0.88)	0.14*** (0.10–0.20)	0.67 (0.31–1.47)	0.64 (0.19–2.19)	0.11*** (0.08–0.13)	0.88 (0.52–1.49)	0.88 (0.40–1.97)
Commerce and sales, marketing	0.07*** (0.06–0.09)	1.60*** (1.15–2.24)	1.60** (1.05–2.45)	0.37*** (0.31–0.46)	0.89 (0.67–1.19)	0.60** (0.40–0.91)	0.21*** (0.18–0.24)	1.10 (0.87–1.40)	0.97 (0.70–1.33)
Engineering, R&D, education	0.14*** (0.11–0.18)	1.18 (0.84–1.67)	1.38 (0.91–2.09)	0.26*** (0.18–0.38)	1.07 (0.62–1.87)	1.10 (0.55–2.19)	0.40*** (0.35–0.47)	1.42*** (1.12–1.80)	1.08 (0.79–1.46)
Other	0.24*** (0.20–0.30)	1.17 (0.91–1.50)	1.49*** (1.13–1.96)	0.13*** (0.10–0.18)	1.86*** (1.30–2.67)	2.96*** (2.01–4.36)	0.47*** (0.41–0.55)	1.11 (0.92–1.35)	1.32*** (1.07–1.63)
Company size (Ref: More than 500 employees)									
1–9 employees	0.93 (0.83–1.05)	1.02 (0.85–1.22)	1.15 (0.92–1.45)	0.97 (0.81–1.16)	0.54*** (0.41–0.71)	1.38 (0.98–1.96)	1.46*** (1.32–1.63)	1.20** (1.01–1.42)	0.97 (0.79–1.18)
10–49 employees	1.12** (1.01–1.25)	1.05 (0.89–1.23)	1.15 (0.94–1.41)	1.09 (0.92–1.29)	0.71*** (0.56–0.92)	1.23 (0.89–1.70)	1.26*** (1.14–1.39)	1.00 (0.86–1.17)	0.96 (0.80–1.15)
50–499 employees	1.31*** (1.20–1.43)	1.07 (0.93–1.23)	0.94 (0.78–1.12)	1.44*** (1.26–1.66)	0.70*** (0.56–0.86)	0.88 (0.67–1.18)	0.99 (0.91–1.07)	1.15** (1.00–1.31)	0.76*** (0.64–0.90)
Year	0.05*** (0.03–0.07)	1.38 (0.91–2.10)	0.74 (0.41–1.36)	0.02*** (0.01–0.03)	1.82** (1.01–3.26)	1.03 (0.44–2.40)	0.14*** (0.09–0.21)	0.75 (0.51–1.09)	0.40*** (0.24–0.68)
Correlation coef.	0.12			0.15			0.21		
No. of obs.	110,824			110,379			111,577		

**, ***The significance level at 5% and 1%, respectively.

a Odds ratio adjusted on all the variables in the model, including the geographical location of the company (region).

b Workers with specific status are employees working in government-owned or controlled corporations and who enjoy a special status SNCF (national railways), the RATP (Parisian transport), the electrical and gas companies (EDG-GDF), etc.

**Table 3:** Results from multivariate random effect logit regressions on severe physical constraints (SUMER 2003, 2010 and 2017)

	Handling of heavy loads			Awkward body postures			Arm/hand vibrations		
	Adjusted prevalence odds ratios^a^			Adjusted prevalence odds ratios^a^			Adjusted prevalence odds ratios^a^		
	(95% CI)			(95% CI)			(95% CI)		
	Main effect	Interc.	Interc.	Main effect	Interc.	Interc.	Main effect	Interc.	Interc.
		2010	2017		2010	2017		2010	2017
Men	1.30** (1.20–1.41)	0.82*** (0.72–0.92)	0.91 (0.77–1.07)	0.76*** (0.72–0.80)	1.00 (0.93–1.08)	1.02 (0.93–1.11)	2.81*** (2.36–3.35)	1.29 (0.99–1.69)	1.38 (0.97–1.97)
Age	0.99*** (0.99–1.00)	1.00 (1.00–1.01)	1.00 (0.99–1.01)	0.99*** (0.99–1.00)	1.00 (1.00–1.00)	1.00 (0.99–1.00)	0.99*** (0.98–0.99)	1.00 (0.99–1.01)	0.99 (0.98–1.00)
Job seniority (Ref: 10 years or more)									
<1 year	1.05 (0.92–1.20)	0.87 (0.71–1.06)	0.77 (0.56–1.05)	0.92 (0.84–1.01)	1.00 (0.88–1.14)	0.81** (0.68–0.97)	1.00 (0.82–1.21)	0.86 (0.65–1.14)	0.86 (0.58–1.29)
1–3 years	1.00 (0.91–1.10)	1.03 (0.89–1.19)	0.88 (0.71–1.08)	0.99 (0.94–1.05)	1.02 (0.93–1.11)	0.88** (0.79–0.99)	1.20*** (1.04–1.37)	0.93 (0.76–1.13)	0.90 (0.69–1.18)
4–9 years	1.08 (0.99–1.17)	0.98 (0.87–1.11)	0.89 (0.75–1.04)	1.01 (0.96–1.06)	1.02 (0.95–1.10)	0.96 (0.88–1.05)	1.19*** (1.06–1.34)	0.89 (0.75–1.06)	0.93 (0.75–1.15)
Full-time	1.29*** (1.17–1.44)	0.92 (0.79–1.08)	1.31** (1.04–1.65)	1.12*** (1.05–1.19)	1.04 (0.95–1.13)	1.05 (0.94–1.18)	1.60*** (1.27–2.02)	1.03 (0.74–1.42)	0.66 (0.44–1.01)
Shift work	1.42*** (1.31–1.53)	0.87** (0.78–0.98)	0.90 (0.77–1.06)	0.88*** (0.83–0.93)	1.03 (0.95–1.12)	1.23*** (1.10–1.36)	0.64*** (0.57–0.73)	0.89 (0.74–1.08)	1.36*** (1.08–1.74)
Night work	1.03 (0.95–1.11)	0.98 (0.87–1.11)	1.02 (0.87–1.21)	1.00 (0.95–1.06)	0.91** (0.84–0.99)	0.88** (0.80–0.98)	0.86** (0.77–0.97)	1.17 (0.97–1.41)	1.06 (0.83–1.33)
Sunday work	1.08** (1.00–1.16)	1.00 (0.89–1.11)	0.73*** (0.63–0.85)	0.92*** (0.88–0.97)	0.97 (0.91–1.05)	1.02 (0.94–1.12)	0.75*** (0.67–0.85)	0.89 (0.75–1.06)	1.70*** (1.37–2.12)
Employment contract (Ref: Permanent contract)									
Agency workers.fixed-term contract	1.03 (0.92–1.16)	0.92 (0.78–1.09)	0.87 (0.67–1.13)	0.99 (0.91–1.07)	0.98 (0.87–1.10)	1.10 (0.94–1.28)	1.06 (0.90–1.25)	1.02 (0.81–1.29)	1.07 (0.76–1.51)
Workers with a specific status^b^	1.70*** (1.47–1.96)	0.76*** (0.63–0.93)	0.23*** (0.15–0.36)	1.15*** (1.04–1.26)	0.78*** (0.69–0.88)	0.78*** (0.67–0.92)	1.68*** (1.14–2.47)	0.77 (0.49–1.21)	0.63 (0.37–1.08)
Occupational status (Ref: Technicians and associate professionals)									
Executives, managers	0.16*** (0.11–0.23)	1.52 (0.96–2.43)	1.92*** (1.14–3.24)	0.97 (0.90–1.04)	1.12** (1.01–1.23)	0.82*** (0.73–0.92)	0.24*** (0.13–0.42)	0.83 (0.37–1.83)	1.16 (0.53–2.52)
Clerks	1.23** (1.02–1.47)	1.37** (1.03–1.81)	0.92 (0.55–1.52)	1.29*** (1.20–1.39)	1.03 (0.92–1.14)	0.90 (0.78–1.04)	—– ——	—— ——	—– ——
Service workers	4.22*** (3.77–4.73)	0.85 (0.72–1.01)	0.97 (0.77–1.22)	1.45*** (1.34–1.56)	0.84*** (0.75–0.93)	1.30*** (1.15–1.48)	1.93*** (1.43–2.59)	0.90 (0.59–1.38)	0.62 (0.36–1.06)
Skilled blue-collar workers	2.41*** (2.16–2.70)	1.04 (0.88–1.23)	1.18 (0.95–1.46)	1.57*** (1.46–1.68)	0.92 (0.83–1.02)	1.73*** (1.54–1.95)	3.16*** (2.74–3.65)	1.05 (0.84–1.30)	1.19 (0.92–1.53)
Unskilled blue-collar workers	4.45*** (3.95–5.02)	0.91 (0.76–1.09)	0.94 (0.73–1.20)	1.59*** (1.47–1.73)	0.91 (0.81–1.02)	1.49*** (1.28–1.73)	2.97*** (2.53–3.50)	1.09 (0.86–1.39)	1.01 (0.75–1.38)
Main occupational duties (Ref: Production, manufacturing and construction)									
Installation, repair, maintenance	0.55*** (0.50–0.62)	0.96 (0.80–1.14)	1.02 (0.80–1.31)	1.66*** (1.54–1.78)	0.96 (0.86–1.08)	0.93 (0.81–1.08)	1.58*** (1.42–1.76)	0.92 (0.79–1.07)	1.25 (1.02–1.53)
Cleaning, childcare. home management	0.38*** (0.32–0.46)	0.82 (0.64–1.05)	0.98 (0.69–1.39)	0.70*** (0.62–0.78)	1.19** (1.02–1.39)	1.32*** (1.08–1.61)	0.61*** (0.46–0.80)	0.99 (0.68–1.44)	1.13 (0.68–1.85)
Handling, logistics, warehousing	2.32*** (2.12–2.55)	0.88 (0.76–1.00)	0.87 (0.72–1.06)	0.88*** (0.81–0.95)	1.03 (0.91–1.16)	1.14 (0.98–1.33)	0.06*** (0.04–0.08)	0.90 (0.56–1.47)	0.94 (0.49–1.83)
Administrative assistant, typing, receptionist	0.14*** (0.10–0.19)	0.41*** (0.24–0.69)	0.28** (0.10–0.81)	0.84*** (0.76–0.94)	1.36*** (1.17–1.58)	1.21** (1.00–1.46)	—– —–	—— —–	—– —–
Management and accounting	0.17*** (0.13–0.22)	0.18*** (0.08–0.41)	0.09** (0.01–0.67)	0.80*** (0.73–0.88)	1.56*** (1.36–1.78)	1.29*** (1.09–1.54)	0.05*** (0.02–0.11)	1.10 (0.27–4.47)	1.01 (0.12–8.52)
Commerce and sales, marketing	0.57*** (0.50–0.65)	0.93 (0.76–1.13)	0.79 (0.61–1.04)	0.61*** (0.56–0.66)	1.45*** (1.29–1.63)	1.30*** (1.12–1.50)	0.08*** (0.05–0.11)	0.78 (0.43–1.39)	0.91 (0.43–1.95)
Engineering, R&D, education	0.09*** (0.05–0.14)	1.04 (0.48–2.22)	1.49 (0.62–3.56)	0.95 (0.87–1.05)	1.18*** (1.03–1.36)	1.09 (0.93–1.28)	0.05*** (0.03–0.10)	0.87 (0.28–2.71)	1.84 (0.62–5.50)
Other	0.74*** (0.63–0.86)	0.84 (0.69–1.02)	0.78** (0.61–0.99)	0.55*** (0.50–0.61)	1.47*** (1.30–1.66)	1.80*** (1.57–2.06)	0.17*** (0.11–0.25)	0.93 (0.57–1.52)	1.35 (0.81–2.25)
Company size (Ref: More than 500 employees)									
1–9 employees	0.92 (0.82–1.04)	1.00 (0.83–1.21)	1.03 (0.81–1.31)	1.01 (0.94–1.08)	0.95 (0.86–1.05)	1.13** (1.00–1.28)	1.32*** (1.08–1.62)	1.34 (0.99–1.83)	1.21 (0.82–1.79)
10–49 employees	1.29*** (1.16–1.44)	0.97 (0.82–1.15)	0.84 (0.68–1.04)	0.98 (0.92–1.05)	0.99 (0.90–1.08)	1.22*** (1.09–1.36)	1.40*** (1.16–1.69)	1.15 (0.87–1.54)	1.17 (0.81–1.67)
50–499 employees	1.37*** (1.25–1.50)	1.02 (0.88–1.19)	0.85 (0.70–1.03)	0.97 (0.92–1.03)	1.08 (0.99–1.17)	1.13** (1.02–1.24)	1.09 (0.94–1.27)	1.25 (0.99–1.58)	1.35 (1.02–1.79)
Year	0.04*** (0.03–0.06)	1.13 (0.77–1.65)	1.03 (0.58–1.82)	0.67*** (0.56–0.80)	1.15 (0.92–1.44)	0.92 (0.68–1.24)	0.02*** (0.01–0.03)	0.68 (0.35–1.30)	0.74 (0.31–1.80)
Correlation coef.	0.09			0.02			0.16		
No. obs.	109,297			109,616			94,273		

**, *** The significance level at 5% and 1%, respectively.

a Odds ratio adjusted on all the variables in the model, including the geographical location of the company (region).

b Workers with specific status are employees working in government-owned or controlled corporations and who enjoy a special status SNCF (national railways), the RATP (Parisian transport), the electrical and gas companies (EDG-GDF), etc.

By contrast, the decrease in exposures to CMR agents, harmful noise and handling of heavy loads occurred to the detriment of other groups of workers. The overexposure of men to CMR agents observed in 2003 worsened in 2017. Differences in exposures to harmful noise and the lifting of heavy loads between full-time and part-time workers also increased over this period. Similarly, all other things being equal, blue-collar workers were exposed more to harmful noise and CMR chemicals than the other socio-professional categories in 2003. However, this gap widened further in relative terms in 2010 and in 2017. In addition, service workers, who had a lower prevalence of exposure to CMR agents than technicians and associate professionals in 2003, were more exposed in 2017 than the latter group. The decline in the exposure to carrying of heavy loads was lower among managers, although they remained the category with the lowest exposure to this occupational risk in 2017.

The general increase over the period 2003–17 in the prevalence of exposures to awkward postures was also heterogeneous across the various socio-professional categories. Compared with technicians, blue-collar and service workers already had a higher probability in 2003 of being exposed to awkward postures, and this situation was more pronounced in 2017. While managers had a probability of exposure to awkward postures that was statistically similar to that for technicians in 2003, they were exposed less often in 2017.

A number of other effects were reversed between 2003 and 2017. Shift work, which was associated with a lower prevalence of exposures to vibrations and awkward postures in 2003, was associated with a higher prevalence in 2017. Similarly, all other things being equal, regular Sunday workers were less exposed to vibrations in 2003 while they were more exposed in 2017. On the other hand, night workers have been under-exposed to awkward postures since 2010. At the company level, size, which was not statistically significant in 2003 and 2010 for exposure to awkward postures, became significant in 2017. Large companies (500 employees or more) have been more successful at limiting exposure to these postural constraints.

## Discussion

The decrease in occupational exposures to factors generating a hazardous physical environment (e.g. CMR agents, harmful noise and extreme temperatures) between 2003 and 2017 in France occurred in the context of tighter regulations. For example, the 23rd of December 2003 Decree on the prevention of chemical risks fundamentally changed the rules by imposing the requirement to perform prior risk assessments and regular measurements of concentrations, and for employees to be informed by their occupational physician of the risks of exposure to CMR agents. Similarly, the 9th of February 2006 Decree established Binding Occupational Exposure Limit Values (BOELVs) for certain CMR agents (e.g. wood dust, benzene, diethylamide and lead). Thus, the safe limit values are an important part of broader risk prevention strategies, and they have the advantage of providing a benchmark for the minimal level in regard to health protection. Awareness of the risks of occupational exposure to CMR agents has also been heightened by the implementation on the 1st of January 2007 of the REACH regulations for streamlining restrictions and for improvement of the regulatory framework of the European Union in regard to chemicals. Moreover, this decrease can also be explained by changes in production processes or by substitution with safer products.

The same observation can be made regarding the decrease in exposure to harmful noise. A major effort to raise awareness of the harmful effects of noise in the workplace has been underway since the early 2000s, with, for example, the ‘European Year of Noise’ organized by the European Agency for Health and Safety at Work in 2005. From a regulatory standpoint, a new European directive, known as ‘Noise’ (2003/10/EC), introduced in 2003, was transposed into French law by decree 2006-892 in July 2006. This regulation defines exposure thresholds above which various preventive actions are mandatory for the employer (e.g. implementation of a program of actions to reduce noise exposure, identification of noisy areas and limitation of their access, use of personal noise protection devices) and, like the exposure limit values for chemical agents, it sets a binding exposure limit value (87 dB once the attenuation provided by personal protective equipment is taken into account).^10^ Nevertheless, in all cases, regardless of the noise level, the directive requires companies to eliminate the risk at the source or to reduce it to a minimum if it is not possible to eliminate it by choosing the least noisy equipment and production processes, by limiting the propagation of noise by enclosing machinery, by enclosing workspaces, or by soundproofing. By contrast, there is no indication in the French Labor Code of the maximum/minimum temperature beyond which it is dangerous or prohibited to work. The employer is merely required to implement the measures necessary to ensure the safety of the employees and to protect the physical and mental health of workers (art. L.4121 of the Labor Code) by the application of general prevention strategies. However, ongoing issues relating to climate change have reinforced the commitment of the labor force to reduce the risks associated with thermal environments. This could explain part of the decline in exposure to these risk factors. It should also be noted that the workforce of some occupations that require working in high heat environments (e.g. dyeing plants, dry cleaners, laundries, blast furnaces, and foundries) decreased over our study period. (In the SUMER survey, the proportion of workers in foundries (in laundries) decreased by 79% (53%) between 2003 and 2017. Nevertheless, a degree of caution is warranted due to the limited number of interviewed workers in these occupations in the survey: approximately 50 in 2003 and 20 in 2017.)

The sharp drop in exposure to repeated handling of heavy loads (10 h/week or more) is undoubtedly largely attributable to technical developments, with mechanized assistance increasingly being adapted to the tasks to be performed.^11^ But, these developments, as well as the preventive measures gradually implemented in companies, have mainly led to a reduction in exposure durations rather than the elimination of the carrying of heavy loads. It must be pointed that in recent years, the handling sector in France has experienced a very strong growth (+13.6% in 2017), with a high demand for automated logistics platforms and warehouses enabling multi-channel distribution in order to satisfy the increasingly short delivery times required by consumers.

The upward trend in exposure to awkward postures is, however, the most worrisome. It is partly due to an increase in the proportion of companies with fewer than 500 employees between 2003 and 2017 in France, coupled with an increase in the prevalence of exposure (+9 pp) in these companies, which have smaller financial resources to implement collective and preventive adaptation policies in regard to occupational health. Yet, severe physical constraints often contribute to the development of MSDs, herniated disks, low back pain and traumatic accidents, which can be a source of physical disability.^12^ The extensive epidemiological literature on this topic shows that working in awkward positions is associated with bruises, sprains, fractures, dislocations and epicondylitis, as well as chronic back, shoulder and neck pain.^12–17^ As exposure to severe physical constraints has not decreased drastically in recent decades and, therefore, remained high (it now affects almost half of all employees), MSDs are still by far the primary cause of compensated occupational diseases. Having steadily increased since the early 1990s, MSDs of limbs and low back pain accounted for 87% of all recognized occupational diseases in 2017. MSDs were also the leading cause of lost working days due to work stoppages, with the loss of more than 10 million working days in 2017 and a direct cost to companies of almost €2 billion for just the MSDs recognized as occupational diseases.^18^ Our analysis of trends in exposure indicates that there is a need to develop new/reinforced interventions to reduce the prevalence of severe physical constraints that contribute to MSDs. Yet at the time that MSDs are placing a huge economic burden on employers and the public healthcare system, France amended the law that defined the list of hardship factors that allowed exposed employees to retire earlier or with better compensation and for which companies had assessment and prevention obligations: the four factors that were removed in 2017 are the handling of heavy loads, awkward postures, mechanical vibrations and dangerous chemical agents. Future surveys of those risk factors will help to determine the extent to which this reform may have affected the prevention behavior of companies and possibly contributed to more extensive occupational exposures.

The other worrisome trend, highlighted by our study, is the significant increase in a number of exposure inequalities, particularly to the detriment of the blue-collar workers. Indeed, the decrease in the risks of exposure to harmful noise and CMR agents between 2003 and 2017 was much lower for blue-collar workers, while at the same time they experienced a more pronounced increase in exposures to awkward postures. These changes in exposure to arduous physical working conditions, the effects of which may extend beyond the workers’ working lives, contribute to social inequalities in health and life expectancy. Indeed, in France in 2016, a 35-year-old blue-collar worker could expect to live in ‘good health’ (i.e. able to comfortably perform everyday tasks) until the age of 77.6 years compared with 84 years for a manager, that is to say, a difference of 6 years in the onset of a degree of physical disability.^19^ In addition to aiming for a general reduction in exposure to all physically demanding working conditions, future adjustments to the national prevention and protection strategies should, therefore, also seek to reduce disparities that affect vulnerable populations and ensure that improvements in occupational safety and health can benefit all workers. Moreover, our results suggest that it would be important to take gender disparities into account to adapt the responses to demanding working conditions issues, and more specifically raise awareness among women and their employers on the need to improve their postural work. Although women are less often assigned to tasks with high physical requirements, such as the handling of heavy loads or using machines that generate arm/hand vibrations, women are more prone to painful postures than their male counterparts. By contrast, increased vigilance must be exercised with regard to exposures to CMR agents among men, since these exposures have decreased, but to a much lesser extent than among women. This gender contrast may be related to the fact that changes in production processes and substitution with safer products are technically easier for some CMR agents than for others and that men and women are not exposed to the same CMR agents. [In 2017, among the CMR agents identified in the SUMER surveys, the most frequent exposures were diesel engine exhaust and wood dust for men (respective prevalence rates of 6.7% and 3.3%) and formaldehyde for women (0.8%). Yet, diesel exhaust and wood dust are among the CMR agents with the smallest decreases in exposure between 2003 and 2017 (−13% and −20% for these agents versus −31% for formaldehyde).]

Our results from large representative samples of French working populations are of great value to nationwide governmental figures and social partners in their decision-making in regard to the requirements for regulations and the allocation of prevention resources. They promote understanding of how to prioritize the actions to be taken in regard to groups with the worst trends in exposures to occupational risks (e.g. blue-collar workers, and shift/night workers for some risk factor). It is likely that reinforcement of prevention measures for well-identified high-priority targets would help reduce the adverse impacts of demanding physical working conditions and associated MSDs.

## Funding

This study was funded by the French Directorate of Research, Studies and Statistics Coordination (Direction de l’animation de la recherche, des études et des statistiques—DARES) of the French Ministry of Labour, in the framework of funding for ‘Social inequalities in Health’ projects. This research received support from the Chair ‘Prevent’Horizon’, under the aegis of the Risk Foundation in partnership with UCLB, Actuaris, AG2R la Mondiale, G2S, COVEA, Groupama Gan Vie, Groupe Pasteur Mutualité, Harmonie Mutuelle, Humanis Prevoyance, and la Mutuelle Générale. The funding partners had no involvement in the analysis and the interpretation of the data, writing of the manuscript, and the decision to submit the manuscript for publication.

*Conflicts of interest*: None declared.

Key points

- Several health policies for the prevention of exposure to physically demanding working conditions have been implemented in France over the past 20 years.
- While the prevalence of exposure to a hazardous physical environment (harmful noise, extreme temperatures, CMR) decreased between 2003 and 2017, the exposure to severe physical constraints (handling of heavy loads, awkward postures) increased.
- Our study highlights a widening of occupational exposure inequalities, particularly to the detriment of the blue-collar workers.
- Future adjustments to the national prevention and protection strategies should, in addition to achieving a general reduction in exposure to all physically demanding working conditions, aim to reduce the disparities affecting vulnerable populations.
- Monitoring trends in disparities will allow public health policy-makers to identify high-priority targets for prevention in order to reduce the adverse impacts of exposure to occupational risks.
